# Trajectories of Compliance With COVID-19 Related Guidelines: Longitudinal Analyses of 50,000 UK Adults

**DOI:** 10.1093/abm/kaac023

**Published:** 2022-06-27

**Authors:** Liam Wright, Andrew Steptoe, Daisy Fancourt

**Affiliations:** Centre for Longitudinal Studies, Institute of Education, University College London, London, UK; Department of Behavioural Science and Health, University College London, London, UK; Department of Behavioural Science and Health, University College London, London, UK

**Keywords:** COVID-19, Nonpharmaceutical interventions, Compliance, Latent class growth analysis, Growth curve modeling

## Abstract

**Background:**

Governments have implemented a range of measures focused on changing citizens’ behaviors to lower the transmission of COVID-19. While international data shows that compliance did decline from the start of the pandemic, average trends could mask considerable heterogeneity in compliance behaviors.

**Purpose:**

To explore trajectories of compliance with COVID-19 guidelines.

**Methods:**

We used longitudinal data on self-reported compliance from 50,851 adults in the COVID-19 Social Study collected across two waves of the pandemic in the UK (April 01, 2020–February 22, 2021). We modeled typical compliance trajectories using latent class growth analysis (LCGA) and used multinomial logistic regression to examine whether individual personality and demographic characteristics were related to compliance trajectories.

**Results:**

We selected a four-class LCGA solution. Most individuals maintained high levels of compliance and reported similar levels of compliance across the first and second waves. Approximately 15% of participants had decreasing levels of compliance across the pandemic, reporting noticeably lower levels of compliance in the second wave. Individuals with declining compliance levels were younger on average, in better physical health, had lower empathy and conscientiousness and greater general willingness to take risks.

**Conclusions:**

While a minority, not all individuals have maintained high compliance across the pandemic. Decreasing compliance is related to several psychological traits. The results suggest that targeting of behavior change messages later in the pandemic may be needed to increase compliance.

## Introduction

Prior to the full roll-out of a vaccine, government strategies to reduce the spread of COVID-19 focused on changing citizens’ behaviors, for instance via advertising personal hygiene reminders (e.g., washing hands), mandating the wearing of face masks, recommending social distancing in public spaces, and prohibiting household mixing. Where followed, these interventions can reduce the spread of the virus [1]. However, each require voluntary cooperation on behalf of citizens, potentially incurring considerable personal costs. Compliance with these behaviors is high but not complete [2,3]. International data shows that average levels of compliance have declined since the start of the pandemic, though compliance increased somewhat as countries experienced second waves [3,4].

While population-level trends have been mapped, these trends could mask considerable heterogeneity: some individuals may have maintained high levels of compliance, while others may have stopped. Existing evidence from the current and previous pandemics shows that trends in compliance can differ markedly across groups [4–7]. For instance, in the UK, compliance decreased faster among younger age groups over the first 5 months of the COVID-19 pandemic in the UK [7]. Yet, to the best of our knowledge, no research has been carried out looking at individual compliance trajectories across the COVID-19 pandemic. This is a striking gap given that variation in infectiousness can influence how viruses spread [8] and that examining individual compliance trajectories could support the targeting and design of interventions for behavior change.

It is also unclear what factors predicted trajectories of compliance during COVID-19. The COM-B model posits that behavior is determined by subjective and objective capability, opportunity for action, and autonomic and reflective motivation [9]. For instance, compliance with COVID-19 social distancing measures is likely to be influenced by knowledge of―and confidence applying―government guidelines (capability), presence of uncrowded environments and social norms conducive to maintaining distance (opportunity), and perceived personal and public health risks from acquiring or transmitting infection (motivation). The COM-B model has previously been applied in the COVID-19 compliance literature, with studies suggesting that motivational factors, such as personal risk of infection and prosociality, are strong determinants of preventive behavior [10–12]. However, this literature has not examined the role of capabilities, opportunities, and motivational factors in *sustaining* compliance over an extended period of the pandemic.

Therefore, in this study, we used (unbalanced) panel data from 50,000 adults from across two waves of the COVID-19 pandemic in the UK (April 2020–February 2021) to model individual trajectories of (self-reported) compliance with COVID-19 guidelines. We used latent class growth analysis (LCGA) to identify “typical” compliance trajectories [13], and tested how compliance trajectories were related to a variety of demographic, personality trait, and individual risk factors categorized using the COM-B framework.

## Methods

### Participants

We used data from the COVID-19 Social Study; a large panel study of the psychological and social experiences of over 70,000 adults (aged >18) in the UK during the COVID-19 pandemic. The study commenced on March 21, 2020 and involved online weekly data collection for 22 weeks with monthly data collection thereafter. The study is not random and therefore is not representative of the UK population, but it does contain a heterogeneous sample. The sample was recruited using three primary approaches. First, convenience sampling was used, including promoting the study through existing networks and mailing lists (including large databases of adults who had previously consented to be involved in health research across the UK), print and digital media coverage, and social media. Second, more targeted recruitment was undertaken focusing on (i) individuals from a low-income background, (ii) individuals with no or few educational qualifications, and (iii) individuals who were unemployed. Third, the study was promoted via partnerships with third sector organisations to vulnerable groups, including adults with preexisting mental health conditions, older adults, carers, and people experiencing domestic violence or abuse. The study was approved by the University College London Research Ethics Committee (12467/005), and all participants gave informed consent. The study protocol and user guide (which includes full details on recruitment, retention, data cleaning, weighting, and sample demographics) are available at https://github.com/UCL-BSH/CSSUserGuide.

For these analyses, we used data from the 11 months between April 01, 2020 and February 22, 2021. To model nonlinear changes in compliance trajectories we focused on individuals with compliance data from three or more data collections across the study period (*n* = 50,851). This sample represents 71.2% of those with data collection by February 22, 2021. Lockdown measures were first announced in the UK on March 23, 2020. The study period overlaps with two waves of COVID-19. Government guidelines and the severity of lockdown measures changed frequently across the study period with some geographic variation. For instance, face masks were mandated in most indoor public places in England and Scotland from mid-August onwards, but in Wales from mid-September. Household mixing rules also differed over time and across regions (see [14] for a summary of policy changes). Supplementary Figure 1 shows 7-day COVID-19 caseloads and confirmed deaths, along with the Oxford Policy Tracker [15], a numerical summary of policy stringency, over the study period.

### Measures

#### Compliance with COVID-19 guidelines

Compliance with guidelines was measured at each data collection using a single-item measure: “Are you following the recommendations from authorities to prevent spread of Covid-19?” The item was measured on a 7-point Likert scale (1 = “Not at all,” 7 = “Very much so”), and analyzed as a continuous variable.

#### Predictors of compliance

We assessed the role of several predictors of compliance. We included variables for demographic and socioeconomic characteristics, social and prosocial factors, physical and mental health, and personality traits, selecting these variables using the COM-B framework of health behavior [9].

For capability to comply, we included variables for locus of control, resilience, educational level, diagnosed psychiatric condition, and the Big-5 personality trait, conscientiousness. We hypothesize that locus of control, resilience, and diagnosed psychiatric condition act as sources of perceived capability, and conscientiousness and education level engender psychological skills enabling sustained compliance. For opportunity to comply, we included variables for country of residence, ethnicity, household income, employment status, neighborhood crowding, and availability of neighborhood space. We hypothesize that ethnicity and country of residence influence compliance through social norms and the other variables influence compliance through changing the physical environment (e.g., difficulty in comfortably maintaining social distancing in the case of neighborhood crowding or increased likelihood of residing in good quality housing―and thus being able to easily remain indoors―in the case of household income).

For motivation to comply, we included variables for long-term physical health conditions (0, 1, and >2), age (grouped), sex, self-isolation status during first the first wave, remaining Big-5 personality traits (openness, extraversion, agreeableness, and neuroticism), (cognitive and emotional) empathy, neighborhood social capital, attachment to neighborhood, neighborhood satisfaction, risk-taking behavior, household overcrowding (>1 persons per room), living arrangement (alone, not alone without child, and not alone with child), and mental health experiences during the first lockdown (same, better or worse vis-à-vis prior to the pandemic). We hypothesized that these variables were related to perceptions or attitudes to risk (long-term physical health conditions, self-isolation, sex [16], neuroticism, and risk-taking behavior), personal emotional or social costs of compliance (living arrangement, overcrowding, mental health experiencing, and extraversion), and prosocial motivations (empathy, social capital, neighborhood attachment, neighborhood satisfaction, and agreeableness).

Several of the variables we used were measured in one-off modules during follow-up and so are missing for many individuals. More detail on the individual measures is provided in the Supplementary Information. These variables have been studied previously in analyses of the COVID-19 Social Study [7,12].

### Statistical Analysis

Our analysis proceeded in three steps. First, we estimated a growth curve model to examine between-person variation in compliance trajectories. To allow for compliance to change nonlinearly with time, we modeled growth curves using natural cubic splines (three degrees of freedom) and included random intercepts and random slopes in this model. Second, we used LCGA to identify “typical” compliance trajectories [13], again using natural splines to allow for flexible relationships with time. We repeated LCGA models for 2–7 classes, using a thresholds link function to account for the nonnormality of our compliance measure. We selected the final model considering the Bayes Information Criterion and entropy values, average latent class probabilities and substantive interpretation of the classes identified. To reduce the risk of the algorithm identifying a local maximum, we fit models with 100 random starts (30 iterations each).

Third, we used multinomial logistic regression to identify predictors of class membership. For each variable, we first estimated a bivariate model and then estimated a multivariate model that included adjustment for sex, country, shielding, psychiatric diagnoses and long-term conditions, household overcrowding, living arrangement, income, (baseline) employment status, ethnic group, education, age group, and Big-5 personality traits [17]. To account for uncertainty in the LCGA classes, we estimated multinomial regressions using “pseudo” draws from posterior probability matrix [18]. We combined this procedure with multiple imputation (*m* = 60) to account for item missingness, pooling estimates using Rubin’s Rules [19]. We used unweighted data to estimate growth curve and LCGA models but added weights in multinomial models. The weights were created using entropy balancing according to population proportions for age, gender, ethnicity, education, and country of living [20]. The data used to create these weights were missing for 357 participants, so the sample is slightly smaller (0.7%) for the multinomial logit models (*n* = 50,494).

Data analysis was carried out in R v 4.0.3. [21]. The growth curve model was estimated using the lme4 package [22], LCGA models were estimated using the lcmm package [23], multinomial regression was carried out the nnet package [24], and imputed data was generated using the mice package [25]. Due to stipulations set out by the ethics committee, data will be made available at the end of the pandemic. The code to replicate the analysis is available at https://osf.io/hmn9s/.

### Role of the Funding Source

The funders had no final role in the study design; in the collection, analysis, and interpretation of data; in the writing of the report; or in the decision to submit the article for publication. All researchers listed as authors are independent from the funders, and all final decisions about the research were taken by the investigators and were unrestricted.

## Results

### Descriptive Statistics

Sample descriptive statistics are displayed in Table 1. Average personality trait levels by socioeconomic groups are shown in Supplementary Fig. 2. Supplementary Table 1 shows descriptive statistics by last month of (continuous) follow-up or whether the participant was ineligible for inclusion in the study. There is evidence of differences in attrition rates across groups. Note, older individuals were more likely to remain in the study. Supplementary Figure 3 shows trends in compliance by the last month of follow-up. Those with higher compliance levels were more likely to remain in the study, but there were qualitatively similar trends in compliance across groups: average compliance decreased from the first lockdown to early Autumn before increasing as the UK faced its second wave.

**Table 1.** Descriptive Statistics. For Continuous Variables, Mean (*SD*). For Categorical Variables, *n* (%)

	Variable	Unweighted observed	Weighted imputed data	Missing %
	*n*	50,851	50,494	
Age (grouped)	18–29	3,674 (7.23%)	9,843.37 (19.49%)	0%
	30–45	13,576 (26.7%)	13,177.79 (26.1%)	
	46–59	16,495 (32.44%)	12,171.91 (24.11%)	
	>60	17,106 (33.64%)	15,300.92 (30.3%)	
Gender	Male	12,406 (24.5%)	24,936.48 (49.39%)	0.43%
	Female	38,228 (75.5%)	25,557.52 (50.61%)	
Ethnicity	White	48,383 (95.46%)	44,032.83 (87.2%)	0.32%
	Non-White	2,303 (4.54%)	6,461.17 (12.8%)	
Country	England	41,148 (80.92%)	42,557.58 (84.28%)	0%
	Wales	5,938 (11.68%)	2,383.76 (4.72%)	
	Scotland	3,239 (6.37%)	4,139.31 (8.2%)	
	Northern Ireland	526 (1.03%)	1,413.35 (2.8%)	
Education	GCSE or below	6,995(13.76%)	16,501.88 (32.68%)	0%
	A-Level	8,820 (17.34%)	17,107.77 (33.88%)	
	Degree or above	35,036 (68.9%)	16,884.36 (33.44%)	
Employment status	Retired	12,224 (24.04%)	10,780.01 (21.35%)	0%
	Employed	31,663 (62.27%)	28,525.58 (56.49%)	
	Student	1,599 (3.14%)	4,500.90 (8.91%)	
	Unemployed/inactive	5,365 (10.55%)	6,687.51 (13.24%)	
Household income	<£16k	6,695 (14.54%)	10,419.08 (20.63%)	9.45%
	£16k–£30k	11,133 (24.18%)	13,820.49 (27.37%)	
	£30k–£60k	16,186 (35.15%)	16,303.94 (32.29%)	
	£60k–£90k	7,131 (15.49%)	6,068.08 (12.02%)	
	>£90k	4,899 (10.64%)	3,882.42 (7.69%)	
Living arrangement	Not alone, no child	27,867 (54.8%)	28,695.19 (56.83%)	0%
	Not alone, with child	13,046 (25.66%)	12,762.13 (25.27%)	
	Alone	9,938 (19.54%)	9,036.69(17.9%)	
Overcrowding	<1 persons per room	45,700 (89.87%)	42,293.91 (83.76%)	0%
	>1 person per room	5,151 (10.13%)	8,200.09 (16.24%)	
Lockdown 1.0 mental health	Same	17,236 (59.32%)	28,592.81 (56.63%)	42.86%
	Worse	9,543 (32.84%)	18,030.78 (35.71%)	
	Better	2,277 (7.84%)	3,870.41 (7.67%)	
Shielding (pre-existing condition)	No	41,646 (82.98%)	41,409.78 (82.01%)	1.31%
	Yes	8,540 (17.02%)	9,084.22 (17.99%)	
Psychiatric condition	No	41,522 (81.65%)	40,186.76 (79.59%)	0%
	Yes	9,329 (18.35%)	10,307.24(20.41%)	
Long-term conditions	0	28,079 (58.42%)	29,312.06 (58.05%)	5.48%
	1	12,845 (26.72%)	13,110.64 (25.96%)	
	>2	7,141 (14.86%)	8,071.30 (15.98%)	
	Openness	15.4 (3.29)	14.88 (3.34)	0%
	Conscientiousness	15.93 (2.97)	15.56 (3.1)	0%
	Extraversion	12.91 (4.29)	12.55 (4.32)	0%
	Agreeableness	15.57 (3.06)	15.35 (3.16)	0%
	Neuroticism	11.32 (4.32)	11.58 (4.51)	0%
	Resilience	20.2 (5.17)	19.84 (5.37)	32.42%
	Optimism	19.76 (4.7)	18.73 (4.84)	39.62%
	(External) Locus of control	12.26 (2.64)	12.72(2.76)	39.73%
	Risk-taking	4.39 (2.35)	4.41 (2.4)	50.47%
	Cognitive empathy	18.72 (4.83)	18.03 (4.95)	41.83%
	Emotional empathy	20.77 (4.64)	20.06 (4.84)	42.08%
	Neighborhood social capital	16.95 (3.49)	16.38 (3.66)	48.18%
	Neighbourhood attachment	10.86 (3.25)	10.2 (3.43)	47.83%
	Neighborhood satisfaction	4.08 (0.93)	3.94 (0.98)	47.58%
	Neighborhood space	8.4 (1.15)	8.27 (1.26)	48.41%
	(Low) Neighborhood crowding	6.97 (1.87)	6.9 (1.89)	48.24%

### Growth Curve Modeling

**Fig. 1.**
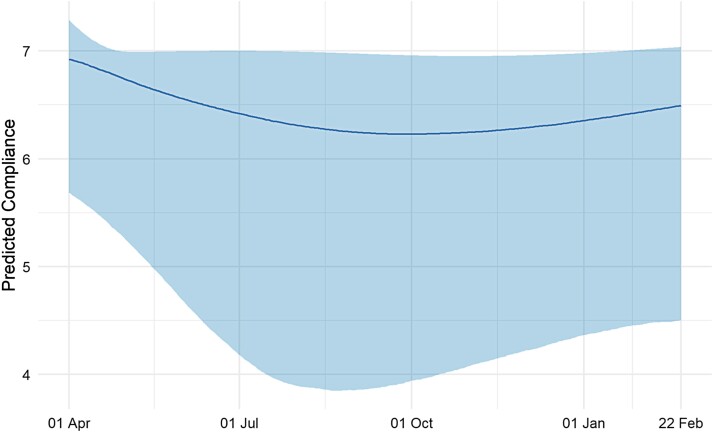
Trends in median compliance level derived from growth curve model with time modeled with natural cubic splines with degrees of freedom 3. Bands represent trends in 2.5th and 97.5th percentiles of predicted compliance levels.

Results from the growth curve model are displayed in Fig. 1. The plot shows trends in the 2.5th, 50th, and 97.5th percentiles of predicted compliance values over the study period. Supplementary Figure 4 plots predicted compliance trends for a random subsample of 6,000 participants in the study. The qualitative trends of declining and then increasing compliance levels are displayed among most individuals but are more pronounced among a set of individuals whose compliance decreases substantially. Among those who compliance declined the most, there was less pronounced increases in compliance during the second wave. For individuals whose compliance decreased to a lesser extent, compliance returned to broadly similar levels as reported in the first wave. The plots demonstrate that population-level trends mask substantial heterogeneity.

### Compliance Trajectory Classes

**Fig. 2.**
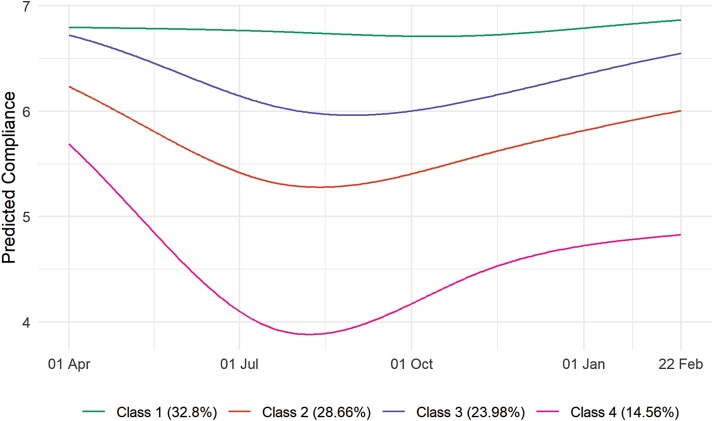
Predicted compliance trajectories by class, four-class LCGA.

Fit statistics from the LGTA models are displayed in Supplementary Fig. 5. We selected a 4-class solution (entropy = 0.82) as solutions with a higher number of classes yielded groups that were similar substantively. Table 2 displays class proportions and average class probabilities. Figure 2 displays predicted compliance trends in each group. Results are alternatively displayed as predicted probabilities in Supplementary Fig. 6 and as a sample of growth curves modeled in the previous section in Supplementary Fig. 7. The largest group (Class 1; 32.8% of weighted observations) consisted of individuals whose compliance remained high throughout the pandemic. Classes 2 and 3 (28.7% and 24%, respectively) consisted of individuals whose compliance was initially high but dropped across summer and increased to (approximately) former levels during the second wave. Class 4 (14.6%), on the other hand, consisted of individuals whose compliance decreased sharply over the first lockdown and, while rising during the second wave, did not reach its former levels. It should be noted; however, that among this group compliance during February 2021 was still predicted to be approximately five on a 1–7 scale.

**Table 2.** Class Proportions and Class Probabilities by Most Likely Class, Derived From Four-class LGCA Model

Most likely class	Class proportions		Average class probability			
	Unweighted	Weighted	Class 1	Class 2	Class 3	Class 4
Class 1	34.6%	32.8%	0.906	0.005	0.089	0.000
Class 2	28.1%	28.7%	0.004	0.889	0.063	0.044
Class 3	26.2%	24%	0.063	0.072	0.865	0.000
Class 4	11%	14.6%	0.000	0.077	0.000	0.923

### Predictors of Compliance Trajectories

**Fig. 3.**
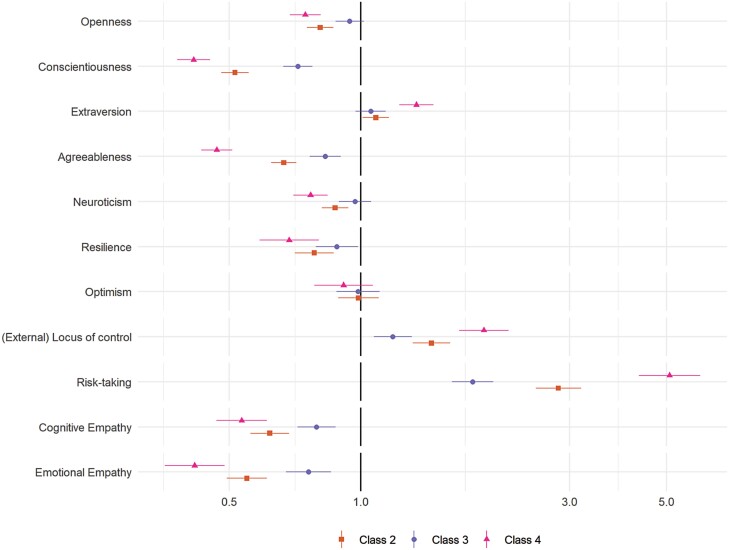
Results of multinomial logistic regression regressing (pseudo-)class membership on personality traits (reference class: Class 1). Adjustment for sex, country, shielding, psychiatric diagnoses and long-term conditions, household overcrowding, living arrangement, income, ethnic group, education, employment status, age group, and Big-5 personality traits. Models use weighted imputed data. Results pooled using Rubin’s [19] rules.

**Fig. 4.**
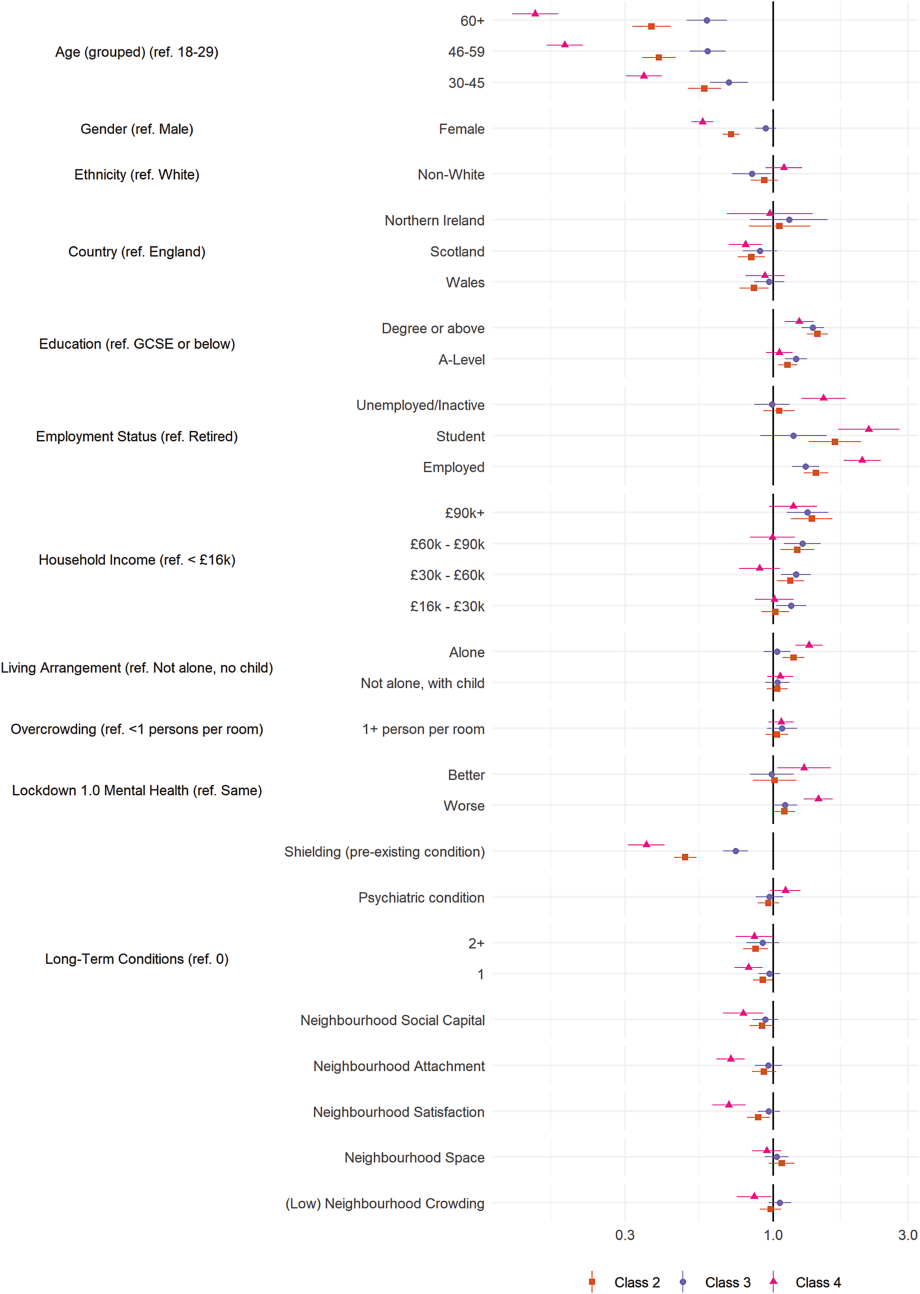
Results of multinomial logistic regression regressing (pseudo-)class membership on demographic, socioeconomic, health and neighborhood factors (reference class: Class 1). Adjustment for sex, country, shielding, psychiatric diagnoses and long-term conditions, household overcrowding, living arrangement, income, ethnic group, education, employment status, age group, and Big-5 personality traits. Models use weighted imputed data. Results pooled using Rubin’s [19] rules.

The results of multivariate multinomial logistic regressions exploring the predictors of class membership are displayed in Fig. 3 (personality traits) and Fig. 4 (demographic, socioeconomic, health, and neighborhood characteristics). (Bivariate regressions are displayed in Supplementary Figs. 8 and 9). For comparability, continuous variables are scaled such that a 1-unit change is equal to a difference of 2 *SD* [26]. Many of the variables were related to compliance trajectories, with several showing strong associations with the low and decreasing compliance pattern (Class 4), including risk-taking behavior, young age, nonretired employment status, (low) emotional empathy and conscientiousness, and shielding due to personal health risk during the first lockdown.

Also related to low and decreasing compliance were (low) resilience, external locus of control, gender (male), low attachment or satisfaction with neighborhood, and trait (low) neuroticism, (low) agreeableness, extraversion, and openness to experience. Note, associations between neighborhood crowding, household overcrowding, or available space and compliance were small, and, relative to stable mental health, both improving and worsening mental health during first lockdown were related to lower compliance.

## Discussion

Using self-report data on compliance with COVID-19 guidelines, we found evidence of substantial heterogeneity in compliance trajectories over the first 11 months of the pandemic in the UK. Across the full sample, average compliance levels decreased only slightly from the first lockdown to mid-Autumn 2020 and returned to similar high levels during the second wave in Winter 2020/2021, but this masked considerable variation: the modal pattern was of consistently high compliance levels, while a minority of individuals showed large decreases in compliance following the first wave which did not fully recover during the second wave. We identified several predictors of compliance trajectories. Note, the low and decreasing compliance pattern was related to age, (baseline) employment status, better physical health, and traits such as risk-taking behavior, low empathy, low conscientiousness, disagreeableness, and low resilience.

The results are consistent with previous work showing declines in average compliance across the pandemic [4], though add richness in suggesting that it is only a minority of individuals for whom compliance substantially decreases. The results suggest that the strongest predictors of low and decreasing compliance were motivational, including risk attitudes and perceptions (proxied by age and physical health) and prosocial motivations, in line with previous studies [10,11,27] and the predictions of the COM-B model. Some authors have argued that low compliance is largely a matter of material difficulties [28], but this is not consistent with the results here nor is it consistent with other studies that have identified several traits―including antisocial dark triad traits―as predictors of compliance [29,30]. Our results indicate that between person differences in compliance are not stable across pandemics and suggest that individual characteristics may become relatively more important in particular contexts [7,30]―here, differences were largest before the beginning of the second wave.

The low and decreasing compliance pattern has implications for discussions that have occurred around “behavioral fatigue,” understood here as a loss of motivation to comply as pandemic progress, holding other things (such as background risk of infection) constant [4,31]. At the beginning of the pandemic, lead UK Government scientists cited behavioral fatigue as a reason to delay the imposition of strict lockdown [32]. The concept was criticized as being poorly elucidated and lacking scientific basis by some groups of behavioral scientists [28,33–37], but has received relatively little testing to date [4,38,39]. Our findings show that some individuals do exhibit a decreasing compliance pattern, and echo other recent work showing declines in population-level compliance [4], between-person differences in compliance motivations [38], and the role of related factors, such as boredom, in predicting compliance behavior [39].

Nevertheless, our results are not dispositive of behavioral fatigue as several factors have changed across the pandemic that could also explain results. One alternative explanation for reduced compliance is “alert fatigue” [40]. Qualitative studies report that individuals have found it difficult to follow frequently-changing government rules [41–43], leading to inadvertent noncompliance as well as bending of rules. However, during the national lockdown in early 2021, rules were simplified and made largely uniform. Furthermore, it is difficult to explain the predictive role of traits such as risk-taking if decreasing compliance was driven by alert fatigue. Other explanations are also possible, notably changes in perceptions of risk. While we are unable to assess this directly, we note that death rates were higher in the second wave (Supplementary Fig. 1). However, subjective risk perception may have reduced regardless, particularly for individuals who believed they had the virus already. Disentangling changes in risk perception from behavioral fatigue is complicated by the possibility that reduced information seeking may be a consequence of fatigue [38] and that individuals could employ motivated reasoning when willingness to comply has fallen [44]. Regardless of the underlying cause, our results show heterogeneity in the level and trajectory of compliance behavior. This has implications for transmission modeling, as well as the targeting or design of interventions for those with the lowest compliance. Specifically, the results suggest decreasing compliance may be driven in large part by motivational factors.

While we find that average compliance declined, it should be reiterated that sustained declines were a minority response. The majority of individuals reported high levels of compliance throughout the pandemic and reported similar levels of compliance in the first and second waves. This suggests that compliance can be largely maintained over extended periods and is consistent with population-level data showing high compliance levels [3]. However, three caveats should be noted. First, we used data from a convenience sample of individuals willing to participate―and continue participating in―a study expressly about COVID-19. These individuals are more likely to comply with COVID-19 guidelines than the wider population, so the extent of noncompliance may be underestimated in this study. Participants were also disproportionately females and highly educated―groups that are more likely to adopt preventive behaviors in pandemics [16]. Second, we modeled compliance as changing continuously through time, but individuals could violate guidelines intermittently to combat fatigue (for instance, occasionally meeting friends) [27]. Designs such as qualitative interviewing could be used to assess this possibility. Third, while our measure of compliance was framed in the present tense, it is possible that previous behaviour could influence responses, restricting temporal change. Nevertheless, our results are consistent with other research that has focused on specific behaviours [4].

There were other limitations of our study. We used self-report compliance data with a single-item measure, which is likely to be subject to issues of social desirability and recall bias. Participants may not have been fully knowledgeable of the rules, particularly as the pandemic progressed [42], and misunderstanding is likely to have been higher among certain groups (e.g., males and those with lower socioeconomic position) [45,46]. Differences in understanding may also have attenuated our regression results. As guidelines varied over the pandemic, the behaviors individuals needed to enact to comply with rules differed over time. This may explain some of the temporal patterns, though using a single nonspecific measure of overall compliance has the advantage that compliance could be tracked over a longer period, given changes in the legal status of certain behaviors.

Some of the associations observed in the multinomial logistic regression modeling may be explained by nondifferential measurement error. Attrition from the study meant that extrapolations further into the pandemic were made for many participants and later follow-ups were biased toward those with higher compliance levels. Though, as noted, compliance *trends* were qualitatively similar regardless of number of follow-ups and we suspect nonrandom attrition means our estimates of noncompliance are conservative. Finally, as noted, we were unable to provide a conclusive test of behavioral fatigue. Innovative designs are required to separate fatigue from other alternative explanations.

Nevertheless, this study also had a number of strengths. To the best of our knowledge, this is the first study investigating individual compliance trajectories during the COVID-19 pandemic. The results in this study may have implications for the modeling of the transmission of the virus, as well as raising questions for the targeting and design of behavior change interventions. This study adds to a small literature examining compliance across the current and previous pandemics, showing variations in behavior across time and between groups [4,5,7,47,48].

## Conclusions

While a minority, not all individuals have maintained high compliance across the pandemic. Decreasing compliance is related to several demographic characteristics and psychological traits. Our results suggest that targeting of behavior change messages later in the pandemic may be needed to increase compliance.

## Supplementary Material

kaac023_suppl_supplementary_Material
